# Supplementary material for: EnzML: multi-label prediction of enzyme classes using InterPro signatures
Source: BMC Bioinformatics. 2012 Apr 25;13:61. doi: 10.1186/1471-2105-13-61 (PMC3483700; doi:10.1186/1471-2105-13-61)
Supplement: Addtional file 5 — The Java code to format the data files, evaluate and predict. The file enzml_java_code.tar.gz contains the Java code used to format database data to ARFF and XML formats, to execute cross and train-test (jackknife) evaluations and to record evaluation results to database. More information is included in the readme.txt file and the Javadoc files. The code can be used with a MySQL database. To use a different database software, other JDBC drivers might be required. [file 1471-2105-13-61-S5.gz › java_code/enzml2011/doc/uk/ac/ed/inf/enzml/test/mulan/MulanArffTest.html]

MulanArffTest


---


|  |  |  |
| --- | --- | --- |
| **Overview** \| **Package** \| **Class** \| **Use** \| **Tree** \| **Deprecated** \| **Index** \| **Help** | |  |
| **PREV CLASS**   **NEXT CLASS** | **FRAMES**    **NO FRAMES**     **All Classes** |  |
| SUMMARY: NESTED \| FIELD \| CONSTR \| METHOD | DETAIL: FIELD \| CONSTR \| METHOD |  |


---


## uk.ac.ed.inf.enzml.test.mulan Class MulanArffTest

```
java.lang.Object
  uk.ac.ed.inf.enzml.test.mulan.MulanArffTest
```

---

``` public class MulanArffTest extends java.lang.Object ```

Class

**Version:**
:   24 Feb 2011

**Author:**
:   Luna De Ferrari luna.deferrari-at-ed.ac.uk

---

| **Field Summary** | |
| --- | --- |
| `static java.lang.String` | `arffInstance1` |
| `static java.lang.String` | `arffInstance2` |
| `static java.lang.String` | `arffInstance4` |
| `static java.lang.String` | `arffInstanceAttributeVoid` |
| `static java.lang.String` | `arffInstanceClassAttributeVoid` |
| `static java.lang.String` | `arffInstanceClassVoid` |
| `static java.lang.String` | `ATT1_TO_STRING` |
| `static java.lang.String` | `ATT2_TO_STRING` |
| `static java.lang.String` | `ATT3_TO_STRING` |
| `static java.lang.String` | `ATT4_TO_STRING` |
| `static java.lang.String` | `ATT5_TO_STRING` |


**Constructor Summary**

`MulanArffTest()`


| **Method Summary** | |
| --- | --- |
| `static java.lang.String` | `getExpectedMulanArff1()` |
| `static java.lang.String` | `getExpectedMulanArff2()` |

**Methods inherited from class java.lang.Object**

`equals, getClass, hashCode, notify, notifyAll, toString, wait, wait, wait`

**Field Detail**

### arffInstance1

```
public static final java.lang.String arffInstance1
```

**See Also:**: Constant Field Values

---


### arffInstance2

```
public static final java.lang.String arffInstance2
```

**See Also:**: Constant Field Values

---


### arffInstance4

```
public static final java.lang.String arffInstance4
```

**See Also:**: Constant Field Values

---


### arffInstanceClassAttributeVoid

```
public static final java.lang.String arffInstanceClassAttributeVoid
```

**See Also:**: Constant Field Values

---


### arffInstanceClassVoid

```
public static final java.lang.String arffInstanceClassVoid
```

**See Also:**: Constant Field Values

---


### arffInstanceAttributeVoid

```
public static final java.lang.String arffInstanceAttributeVoid
```

**See Also:**: Constant Field Values

---


### ATT1\_TO\_STRING

```
public static final java.lang.String ATT1_TO_STRING
```

**See Also:**: Constant Field Values

---


### ATT2\_TO\_STRING

```
public static final java.lang.String ATT2_TO_STRING
```

**See Also:**: Constant Field Values

---


### ATT3\_TO\_STRING

```
public static final java.lang.String ATT3_TO_STRING
```

**See Also:**: Constant Field Values

---


### ATT4\_TO\_STRING

```
public static final java.lang.String ATT4_TO_STRING
```

**See Also:**: Constant Field Values

---


### ATT5\_TO\_STRING

```
public static final java.lang.String ATT5_TO_STRING
```

**See Also:**: Constant Field Values


**Constructor Detail**

### MulanArffTest

```
public MulanArffTest()
```


**Method Detail**

### getExpectedMulanArff1

```
public static java.lang.String getExpectedMulanArff1()
```

---


### getExpectedMulanArff2

```
public static java.lang.String getExpectedMulanArff2()
```


---


|  |  |  |
| --- | --- | --- |
| **Overview** \| **Package** \| **Class** \| **Use** \| **Tree** \| **Deprecated** \| **Index** \| **Help** | |  |
| **PREV CLASS**   **NEXT CLASS** | **FRAMES**    **NO FRAMES**     **All Classes** |  |
| SUMMARY: NESTED \| FIELD \| CONSTR \| METHOD | DETAIL: FIELD \| CONSTR \| METHOD |  |


---
